# Cross-cultural differences in self-reported and behavioural emotional self-awareness between Japan and the UK

**DOI:** 10.1186/s13104-023-06660-0

**Published:** 2023-12-21

**Authors:** Charlotte F. Huggins, Justin H. G. Williams, Wataru Sato

**Affiliations:** 1 Translational Neuroscience, Institute of Medical Sciences, School of Medicine, Medical Sciences, and Nutrition, University of Aberdeen, Aberdeen, AB24 3FX UK; 2 Child and Youth Mental health Service, Gold Coast Health Robina Hospital, 2 Bayberry Lane, Robina, QLD 4226 Australia; 3 Psychological Process Research Team, Guardian Robot Project, RIKEN. 2-2-2 Hikaridai, Seika-cho, Soraku-gun, Kyoto, 619-0288 Japan

**Keywords:** Alexithymia, Culture, Emotional awareness, Emotional differentiation

## Abstract

**Objective:**

How we express and describe emotion is shaped by sociocultural norms. These sociocultural norms may also affect emotional self-awareness, i.e., how we identify and make sense of our own emotions. Previous studies have found lower emotional self-awareness in East Asian compared to Western samples using self-report measures. However, studies using behavioural methods did not provide clear evidence of reduced emotional self-awareness in East Asian groups. This may be due to different measurement tools capturing different facets of emotional self-awareness.

**Results:**

To investigate this issue further, we compared the emotional self-awareness of Japanese (n = 29) and United Kingdom (UK) (n = 43) adults using the self-report Toronto Alexithymia Scale (TAS-20), alongside two behavioural measures – the Emotional Consistency Task (EC-Task) and the Photo Emotion Differentiation Task (PED-Task). Japanese adults showed higher TAS-20 scores than UK participants, indicating greater self-reported difficulties with emotional self-awareness. Japanese participants also had lower EC-Task scores than UK adults, indicating a lower ability to differentiate between levels of emotional intensity. PED-Task performance did not show clear group differences. These findings suggest that cross-cultural differences in emotional self-awareness vary with the task used, because different tasks assess distinct aspects of this ability. Future research should attempt to capture these different aspects of emotional self-awareness.

**Supplementary Information:**

The online version contains supplementary material available at 10.1186/s13104-023-06660-0.

## Introduction

Emotion is an integral part of our daily lives, driving socialisation, decision-making and well-being. Emotion is also shaped by culture; that is, the terms we use to describe emotion are learned through social interactions and shaped by cultural evolution [1]. Moreover, not all emotion terms exist in all languages and cultures [1]. Different cultures have different norms about how emotions should be expressed and how desirable certain emotions are considered [2]. Therefore, it appears likely that there may also be cultural differences in how people identify and describe their own emotions.

The ability to identify and describe our own emotions is likely to be fundamental to being able to manage and communicate them to others. However, defining and measuring emotional self-awareness is no easy task. Many constructs have emerged in the literature that attempt to capture emotional self-awareness, all with subtly different definitions and measurement methods [3]. In this study, we use ‘emotional self-awareness’ as an umbrella term to describe the general ability to identify, label and understand one’s own emotions. This term also includes a range of other constructs, such as emotional differentiation, i.e., the ability to differentiate between similar qualitative emotional states [4], and alexithymia [5], a clinical construct describing severe difficulties describing emotions, as well as imaginative deficits. We suggest that these constructs fall under the umbrella of emotional self-awareness, but represent subtly different aspects of this ability, similar to frameworks for the various aspects of interoception [6].

Previous self-report studies have consistently found lower emotional self-awareness in East Asian populations than in Western populations [7–10]. These studies used the Toronto Alexithymia Scale (TAS-20) [11], a self-report questionnaire assessing alexithymia – difficulty identifying and describing one’s own emotions; it is one of the most commonly used tools to assess emotional self-awareness. The results consistently showed elevated TAS-20 levels in the East-Asian population, compared to those with Western backgrounds. However, it has long been suggested that alexithymia is based on Western cultural norms of emotional expression and communication [12]. As such, there may not be a complete conceptual equivalent of alexithymia across cultures [13], as standards of what represents ‘good’ or ‘normal’ emotional communication varies. Moreover, research suggests that cultural differences in alexithymia scores are more strongly driven by subscales assessing ‘externally oriented thinking’ (i.e., the tendency to focus on external stimuli rather than internal sensations), rather than those reflecting the ability to identify one’s own emotions [8].

In contrast, behavioural studies have provided inconsistent findings, comparing emotional self-awareness in East Asian and Western samples. One study, using the Levels of Emotional Awareness Scale (LEAS) [14], found lower scores in Japanese participants than in American participants [15]. The LEAS is a vignette tool that assesses emotional self-awareness through people’s descriptions of their feelings in response to a variety of hypothetical scenarios. However, it must be noted that this earlier study used different scenarios across the samples and tested the samples under different conditions (i.e., individually vs. in a group). Furthermore, responses describing bodily and somatic sensations are scored lower on the LEAS [14]. It has long been suggested that East Asian cultures and languages are more likely to describe emotions in terms of physiological sensations [13]; as such, differences may be due to linguistic and cultural differences, rather than actual ability. Goetz et al. [16] suggested that the greater dialecticism – the tendency to accept contradiction and change – seen in East Asian cultures leads to greater tolerance of more complex and contradictory emotional states. This results in East Asian participants being more likely to report co-occurring positive and negative states. Furthermore, Grossmann et al. [17] found higher emotional differentiation in Japanese compared to American adults. In this study, American and Japanese participants rated their ten recent emotional experiences in terms of how strongly the experiences elicited nine distinct emotional states. Japanese participants showed stronger differentiation between emotional states of the same valence (i.e., lower intraclass correlations of valence-specific ratings), applying them in different ways to the different scenarios, thus indicating better emotional self-awareness. Moreover, Japanese participants were also more likely to experience mixed emotions (i.e., reporting a higher frequency of co-occurring negative and positive emotions). In short, these behavioural studies suggested lower [15] or higher [16, 17] emotional self-awareness in East Asian compared with American participants.

One explanation for these contradictory findings is that the differences may depend on the measure used, as the measures may differ in task demands and tap into different ‘aspects’ of emotional self-awareness. In the abovementioned studies, lower emotional self-awareness in East Asian samples emerged in self-report analyses, but not in behavioural measures. This may also be due to a greater tendency to modesty in East Asian than in Western cultures [18–20], which pertains even in anonymous self-report measures [21]. Such modesty may lead to Japanese samples underreporting their own emotional competence, resulting in elevated TAS-20 scores.

To investigate this issue, we compared the emotional self-awareness of Japanese and United Kingdom (UK) adults using the self-report TAS-20, alongside two behavioural measures, the Emotional Consistency Task (EC-Task) [22] and the Photo Emotion Differentiation Task (PED-Task) [23]. In the EC-Task, participants are presented with pairs of emotional photographs and are instructed to select the one that evokes a stronger emotional experience. The logical consistency of these emotional decisions is examined, generating indices of emotional differentiation. In the PED-Task, participants are presented with individual emotional photographs and are instructed to rate how strongly they feel in terms of 10 emotional states. Correlations between similar emotional states are examined, generating indices of emotional differentiation. The TAS-20 is likely to mirror metacognitive awareness of one’s own emotional abilities. The goal of this study was to examine whether cultural differences in emotional self-awareness are consistent across different measurement tools, in an attempt to evaluate more accurately whether inconsistency in the research literature is related the measurement tool applied. We hypothesised that Japanese participants would show high scores on the TAS-20, but no differences in emotional self-awareness on the EC-Task and PED-Task.

## Main text

Methods are presented in Supplementary Material 1. Code and output for main analyses can be seen in Supplementary Material 2.

## Findings

**Fig. 1:**
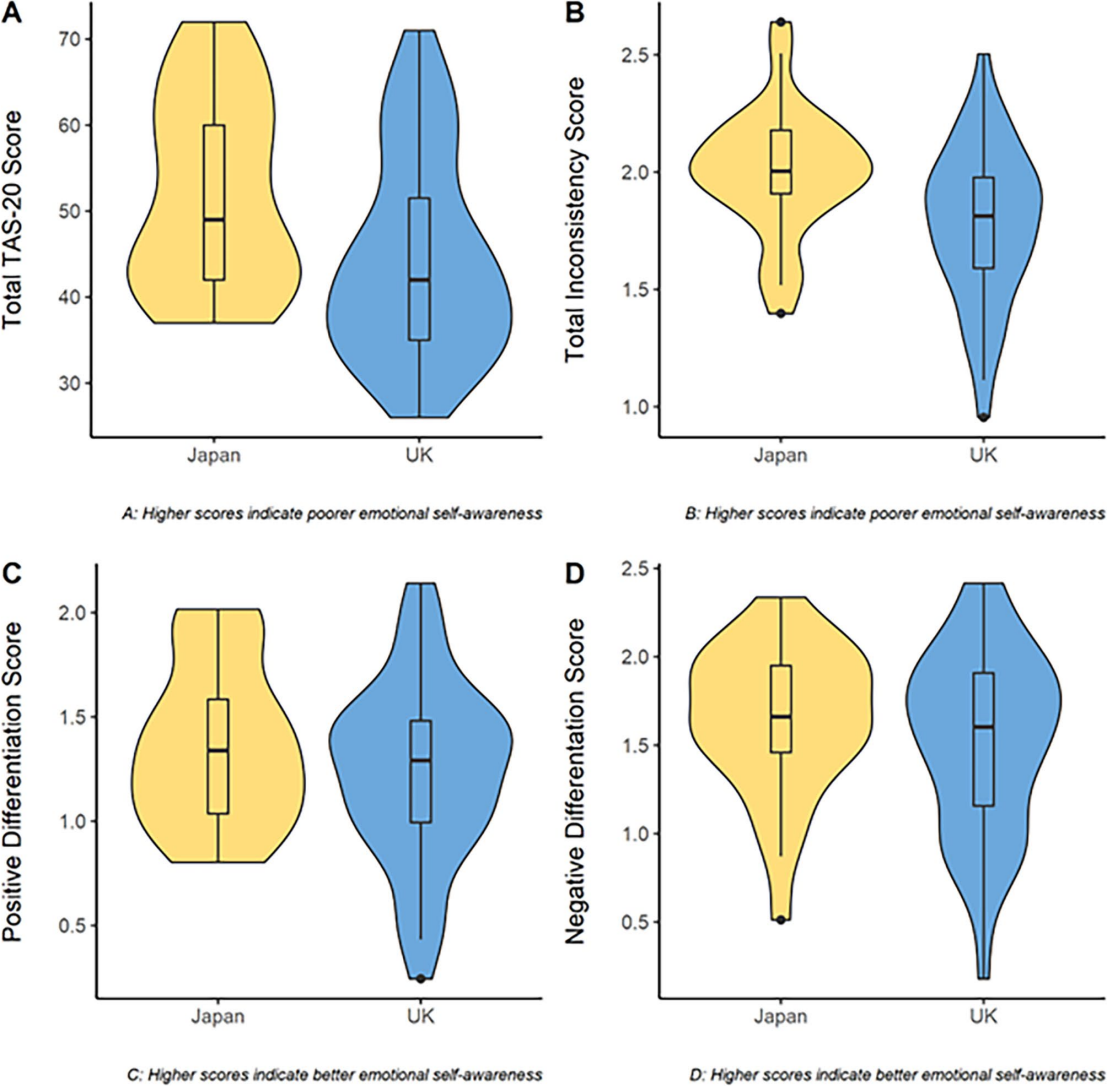
Violin plots of emotional self-awareness measures for Japanese and United Kingdom (UK) participants. TAS-20 = Toronto alexithymia scale

All self-report scores were normally distributed. Table 1 presents the average values of key measures, as well as Welch’s *t*-test comparisons, between cultural groups. Figure 1 shows violin plots of all emotional self-awareness comparisons.

**Table 1:** Mean (with *SD*) scores and statistics for main outcomes among Japanese and United Kingdom (UK) samples

Measure	Japanese (*n* = 29)			UK (*n* = 43)			Statistic (Welch’s *t*-test)
	*M*	*SD*		*M*	*SD*		
TAS-20	51.90	11.17		44.44	11.65		***t*(62.0) = 2.729**, ***p*** **= .008**, ***d*** **= 0.654**
TAS-20 DIF	17.90	6.21		15.30	5.55		*t*(55.6) = 1.814, *p* = .075, *d* = 0.441
TAS-20 DDF	16.17	4.46		12.86	4.84		*t***(63.5) = 2.986**, ***p*** **= .004**, ***d*** **= 0.711**
TAS-20 EOT	17.83	4.62		14.23	3.88		***t*(53.0) = 3.447**, ***p*** **= .001**, ***d*** **= 0.846**
Total Inconsistency	2.00	0.27		1.78	0.33		***t*(67.7) = 3.105**, ***p*** **= .003**, ***d*** **= 0.730**
Positive Differentiation	1.36	0.38		1.24	0.43		*t*(64.5) = 1.291, *p* = .201, *d* = 0.296
Negative Differentiation	1.64	0.42		1.53	0.51		*t*(67.4) = 1.053, *p* = .296, *d* = 0.235

Bold text indicates significant differences at α = 0.05. TAS-20 = Toronto Alexithymia Scale; DIF = Difficulty Identifying Feelings; DDF = Difficulty Describing Feelings; EOT = Externally Oriented Thinking

### Self-reported emotional self-awareness

Welch’s *t*-test found significantly higher TAS-20 scores in Japanese participants (*M* = 51.90, *SD* = 11.17) compared to UK participants (*M* = 44.44, *SD* = 11.65), *t*(62.0) = 2.729, *p* = .002, *d* = 0.650. Linear regression analysis was performed to evaluate whether differences remained significant while controlling for gender and age. Only nationality was significant, β = −0.316, *p* = .011, with neither gender, β = −0.061, *p* = .608, nor age, β = −0.170, *p* = .142, reaching significance. These findings suggest that TAS-20 scores were significantly higher in Japanese compared to UK participants, although the *R*^2^ was low, with the model only accounting for 8.9% of total variance.

Subscales of the TAS-20 were also compared using Welch’s *t*-tests. For the Difficulty Identifying Feelings subscale, Japanese participants (*M* = 16.17, *SD* = 4.46) scored significantly higher than UK participants (*M* = 12.84, *SD* = 4.84), *t*(63.5) = 2.986, *p* = .004, *d* = 0.771, suggesting greater difficulties describing one’s own feelings. A similar effect was found for the Externally Oriented Thinking scale, with Japanese participants (*M* = 17.83, *SD* = 4.62) again scoring higher than UK participants (*M* = 14.23, *p* = 3.88), *t*(53.0) = 3.447, *p* = .001, *d* = 0.857. No difference emerged for the Difficulty Describing Feelings subscale, *t*(55.6) = 1.814, *p* = .070.

### Behavioural emotional self-awareness

Behavioural emotional self-awareness was assessed using the EC-Task and PED-Task. In the EC-Task, Welch’s *t*-test found that Japanese participants (*M* = 2.00, *SD* = 0.27) had significantly higher Total Inconsistency scores than UK participants (*M* = 1.78, *SD* = 0.33), *t*(67.7) = 3.105, *p* = .003, *d* = 0.730. Linear regression analysis, including performance in the non-emotional control condition, gender, and age as covariates, was conducted to assess the significance of these differences. Nationality significantly predicted Total Inconsistency scores, β = -0.441, *p* < .001, with being from the UK associated with more consistent emotional decision-making.

In the PED-Task, initial Welch’s *t*-tests found no differences among cultural groups in terms of positive or negative differentiation, *t*(64.5) = 1.291 and 1.053, *p* = .201 and 0.296, *d* = 0.296 and 0.235, respectively. To confirm this result, a multivariate analyses of covariance (MANCOVA) controlling for age and gender was conducted. MANCOVA was selected so that positive and negative emotion differentiation could be compared in the same analysis. No significant effect for nationality was found, *F*(2, 67) = 0.975, *p* = .382. Likewise, no effects were found for either age, *F*(2, 67) = 2.258, *p* = .113, or gender, *F*(2, 67) = 0.179, *p* = .837.

## Discussion

In this study, we examined potential differences in Japanese and UK participants on three measures of emotional self-awareness. In line with previous research [7, 8, 10, 24], TAS-20 scores were significantly higher in Japanese compared to UK samples, indicating lower emotional self-awareness, which was consistent with our hypothesis. Unexpectedly, the EC-Task performance was also lower in Japanese compared to UK samples, indicating lower emotional self-awareness. As expected, the PED-Task showed no clear difference, indicating no cultural difference in the ability to differentiate between qualitatively similar emotional experiences. Notably, while all three measures capture emotional self-awareness, they have different biases and may reflect different aspects of the same ability.

In contrast to our hypothesis, no evident cultural differences were observed in emotional self-awareness between subjective (i.e., TAS-20) and behavioural (i.e., the EC- and PED-Tasks) measures. However, differences were observed between the former two subjective and behavioural tasks and the latter behavioural task, suggesting that differences in these methods could not accounting for cultural differences in emotional self-awareness. We speculate that different levels of metacognitive self-awareness in the measures may underlie these findings. The TAS-20 requires metacognitive self-awareness of emotional ability (i.e., how people rate their own abilities). However, the PED-Task, in which participants rate their online emotional states in response to individually presented emotional photographs, does not require metacognitive self-awareness. Conversely, the EC-Task, in which participants select the photograph evoking stronger emotions from pairs of repetitively presented emotional photographs, uses similar principles to transitive reasoning [22] and may implicitly activate the metacognitive self-awareness to make consistent decisions. Therefore, stronger metacognitive self-awareness of emotional ability in Western compared to East Asian cultures may produce distinct cultural patterns across the tasks. This speculation is consistent with the previous finding that self-monitoring, which is conceptualized as self-observation and self-control [25] and is related to metacognitive awareness [26], was higher in Western (i.e., United States) compared to East Asian (i.e., Japan) cultures [27]. It was proposed that cultures with high individualism focus on the self, rather than others; in contrast, cultures with high collectivism consider the effects on other individuals when deciding how to behave in a particular situation [25]. We speculate that such cultural differences in metacognitive self-awareness may produce divergent cultural patterns in emotional self-awareness, as assessed by self-reported and behavioural measures, among Western and Eastern cultures. Future studies are needed to test these hypotheses empirically.

### Limitations

A key limitation of our study was the small sample sizes of the UK (*n* = 43) and Japanese (*n* = 29) groups. This constraint raises concerns regarding the generalisability of our findings. A small sample size can lead to several issues, particularly in cross-cultural studies. First, the limited number of participants may not adequately represent the broader populations of Japan and the UK. Cultural diversity within each country means that a small group may not capture the full range of emotional self-awareness present in each culture. Furthermore, small samples limit the power of statistical analyses, making it challenging to draw firm conclusions from the data. Specifically, the lack of significant differences observed in the PED-Task could reflect a lack of power to detect existing differences rather than true similarities between cultures. However, this study was the first to test the same sample with subjective and behavioural tasks and show different patterns across the tasks indicating significant differences in TAS-20 scores and EC-Task performance, but not in PED-Task performance. Thus, our study was a pilot study suggesting that cross-cultural differences in emotional self-awareness may vary with the task used. To validate our findings, future research should investigate larger samples and provide a more reliable and generalisable understanding of the cultural differences in emotional self-awareness.

### Electronic supplementary material

Below is the link to the electronic supplementary material.

Supplementary Material 1

Supplementary Material 2

## Data Availability

The data that support the findings of this study are available onrequest from the corresponding author.
