# Supplementary material for: Cross-cultural differences in self-reported and behavioural emotional self-awareness between Japan and the UK
Source: BMC Res Notes. 2023 Dec 21;16:380. doi: 10.1186/s13104-023-06660-0 (PMC10734098; doi:10.1186/s13104-023-06660-0)
Supplement: Supplementary file 1 — Supplementary Material 1 [file 13104_2023_6660_MOESM1_ESM.docx]

# Additional File 1: Methods

## Participants

Twenty-nine participants (15 men, 14 women) were recruited from Kyoto University. All participants received ¥1,500 (roughly £11) in coupons as compensation for their time. Participants’ age ranged from 19 to 50 years, with a median age of 22 years (interquartile range: 4 years). Data were collected as part of the lead author’s Japanese Society for the Promotion of Science Summer Programme Fellowship, limiting the recruitment period to between June and August 2019. Sample size was determined by an a priori power analysis. G*Power ver. 3.1.9.2 software [1] was used with the intent of comparing groups using independent *t*-tests (two-tailed) for self-reported (i.e., Toronto Alexithymia Scale [TAS-20] scores) and behavioural (i.e., the Emotional Consistency [EC] Task and Photo Emotion Differentiation [PED] Task) emotional self-awareness measures with an α level of 0.05, a power of 0.80, and an effect size *d* of 0.8 (strong; cf., [2]). The results indicated that 26 participants were required for each group.

To act as a comparison group, a subsample of participants recruited in the United Kingdom (UK) were selected from a previous study [3,4] conducted at the University of Aberdeen. Only participants who clearly indicated their native language as English were included. Consequently, the comparison group was composed of 43 participants (10 men, 33 women). All UK comparison participants completed English-language versions of the same tools and were reimbursed for their time with either course credit or £10 cash. Experimental procedures for Japanese participants were kept as close as possible to those of previous UK participants, including the order of tasks, the measures included, and the instructions given. Not all measures used in the study were included in the analysis. Participants in both groups were largely undergraduate students, recruited through convenience sampling at the university.

Informed consent was obtained from all participants. The study protocol was approved by the Ethics Committee of the Unit for Advanced Studies of the Human Mind, Kyoto University, and the University of Aberdeen School of Psychology Ethics Committee. Testing was conducted in accordance with the Declaration of Helsinki.

## Materials

### Japanese Toronto Alexithymia Scale (J-TAS-20)

Self-reported emotional self-awareness was measured with the Japanese 20-item Toronto Alexithymia Scale (J-TAS-20) [5]. The TAS-20 [6], one of the most commonly used measures of alexithymia and emotional self-awareness, has a three-factor structure with three subscales: Difficulty Identifying Feelings, Difficulty Describing Feelings, and Externally Oriented Thinking. Participants respond to each item, which is scored on a 5-point scale and summed to produce total and subscale scores. The J-TAS-20 has the same scoring procedure as the TAS-20 and shows sufficient internal consistency, good retest reliability, and a valid three-factor structure [7]. Several previous studies have supported the generalisability of the three-factor structure of TAS-20 across various Western and Eastern populations [8]. Recent studies further demonstrated (at least partial) measurement invariance between Western and Eastern cultures of TAS -20, indicating its cross-cultural equivalence [9,10].

### Emotional Consistency Task (EC-Task)

The EC-Task is a tool that measures emotional self-awareness through consistency in emotional decision-making [3]. Participants view pairs of emotional images from the Nencki Affective Picture System (NAPS) [11]. For each pair, they choose the image that evokes the stronger emotional response. Whether participants choose based on a stronger positive or negative emotional response depends on the task condition. Here, it was stressed that participants were not choosing based on image quality or how they thought other people might tend to respond to the images but, instead, on the basis of their own personal emotional response to the images.

Emotional self-awareness was quantified by examining how consistently participants chose between the stimuli, with greater consistency reflecting a better ability to differentiate between similar levels of emotional intensity and thus greater emotional consistency. Full details of the task are described in Huggins et al. [3].

The task was divided into five conditions, with 55 pairs of 11 images in each. The first condition was a non-emotional control task, in which participants chose between images based on ‘colourfulness’. Colourfulness was chosen as this was a subjective decision that was not directly related to the emotional qualities of the image. This was followed by the four experimental conditions, in a 2 (valence: positive vs negative) × 2 (difficulty: easy vs hard) design. Images in the experimental conditions all depicted people, e.g., a positive image depicting a group of people enjoying music in the park, or a negative image depicting a paramedic carrying an injured man. Images were selected to depict emotionally evocative situations that adults would be likely to see on the news or in everyday scenarios.

In the positive conditions, all images had an overall positive valence, and participants were asked to choose based on which image was more ‘pleasing’ (快に感じる). In the negative conditions, all images had an overall negative valence, and participants were asked to choose based on which image was more ‘upsetting’ (不快に感じる).

In the easy conditions, images had a wider range of valence ratings, making it easier to choose which image was more emotionally evocative. In difficult conditions, the ratings were more similar, making the task more challenging. Previous work has found that participants tend to show better consistency in easy tasks than in difficult tasks [3], providing evidence of the task’s validity.

The EC-Task yields four scores of emotional consistency (Positive-Easy, Positive-Hard, Negative-Easy, and Negative-Hard), as well as a non-emotional Control score, reflecting general consistency in decision-making. It should be noted the EC-Task shows images to elicit emotional responses in the viewer. The participant then makes a decision based on their own emotional response, rather than any emotion depicted in the images.

Consistency scores were calculated for each set of images. Consistency of decision-making was quantified by comparing the individual decision of a participant made between image pairs to how frequently they chose each image overall. Full details of the consistency calculation are provided in Huggins et al. [3]. More consistent decisions indicate that participants were better able to differentiate between similar levels of emotional arousal. For each condition, there was a maximum possible score of ‘220’, indicating no consistency errors in decision-making. To create total consistency scores, scores from the four emotional conditions were summed, creating a maximum possible score of 880. The Consistency Task and scorer were developed and implemented in MATLAB R2017a (MathWorks, Natick, MA, USA).

As in a previous work using the EC-Task [3], all consistency scores were negatively skewed, and some participants showed perfect consistency in each condition. All consistency scores were log-transformed to fit the assumptions of normality. Following transformation, higher scores reflect poorer consistency and thus poorer emotional self-awareness. For the sake of clarity, these transformed scores are referred to as ‘inconsistency scores’.

### Photo Emotion Differentiation Task (PED-Task)

In the PED-Task [12], participants viewed NAPS images paired with emotion words. They rated how strongly the target image made them feel the target word on a 0 to 6 Likert scale. Twenty images (10 positive; 10 negative) were presented alongside 10 (5 positive; 5 negative) emotion words. Each image was shown with each word once, resulting in 200 trials. These 200 trials were presented in a random order, and participants could take as long as they wanted on each trial. Task procedure and stimuli were identical to Huggins et al. [3]. Details of the terms used are described in Supplementary Material. The PED-Task was implemented in PsychoPy version 3, and scores were calculated with a MATLAB script. Similar to the EC-Task, the PED-Task asks participants to evaluate their own emotional responses to the images, rather than judge the emotions depicted in the images.

Emotional differentiation in the PED-Task was calculated using the mean standard deviation (MSD), rather than intraclass correlation (ICC). The MSD was chosen as the ICC results in uninterpretable scores (e.g., scores below zero) that can result in large amounts of missing data [13], which is unsuitable for such a small sample. The MSD reflects how much the participant varied in applying similar emotional terms to the same stimuli. A low score indicates that the participant applied different emotion terms to the same stimuli in the same way (e.g., responding in a globally negative way to a negative emotion stimulus), indicating low emotional differentiation. A higher score indicates that participants applied emotion terms in a more varied way, indicating high differentiation.

To calculate the MSD, the SDs of the same-valence emotion ratings for each image were first calculated (e.g., how the participant rated positive image A on the five positive emotion terms). Lower SDs indicate that the participant applied the different emotion terms to the same stimuli in a similar way (e.g., rating all images as very positive, regardless of the type of positive emotion invoked). The mean of these SDs were then calculated for images and words of the same emotion type (e.g., positive images with positive words); the formula is given below, in which SDX represents the standard deviation of congruent ratings (i.e, positive emotion terms with positive images) of image X and n represents the number of images in the set.

**
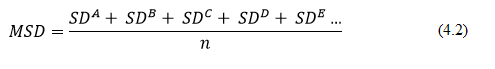
**

This produced two separate indices of negative and positive emotion differentiation, in which higher scores reflected a greater ability to differentiate between similar discrete emotional states, and thus greater emotional self-awareness.

## Translation

Translation of the behavioural tasks was conducted by WS, a bilingual Japanese native, and CFH, an English native speaker with intermediate Japanese proficiency. Instructions and keywords were back translated at least three times to ensure that the meaning was retained. Native Japanese speakers with no prior knowledge of the study read the instructions to confirm that they were understandable, providing feedback on where wording needed to be changed.

## Procedure

The experiment was performed in a small soundproof room under dim lighting. The events were controlled by in-house programs on a Windows laptop computer. The participants were seated comfortably approximately 0.6 m from the monitor. The study was conducted in Japanese by a native speaker experienced in administering psychological experiments. Each participant took part alone and completed each part of the study in the same order. The experimenter first explained the procedure orally and provided written information about the study to the participant. The lead author was on-hand to answer questions if necessary, as well as to respond to any technical difficulties. The participant first took part in the EC-Task, beginning with the colourfulness control condition. This was followed by the working memory task, and then the PED-Task. The participant then completed the battery of self-report questionnaires digitally, including the TAS-20, using a laptop-tablet. The participant was finally debriefed and thanked for their time. The procedure was kept as similar as possible to the testing of UK participants in Huggins et al. [3].

# *Statistical Analyses*

All of the following analyses were conducted in R v3.6.1. Code and output for the main analyses are provided in Supplementary Material. Welch’s *t*-test was conducted to compare cultural groups, as it produces robust results for groups with different sample sizes [14]. Linear regression analysis and multivariate analysis of covariance were conducted to compare groups while controlling for covariates (e.g., gender).References

1. Faul F, Erdfelder E, Lang AG, Buchner A. G*Power 3: A flexible statistical power analysis program for the social, behavioral, and biomedical sciences. Behav Res Methods. 2007;39, 175–91.

2. Igarashi T, Komaki G, Lane RD, Moriguchi Y, Nishimura H, Arakawa H, Gondo M, Terasawa Y, Sullivan CV, Maeda M. The reliability and validity of the Japanese version of the Levels of Emotional Awareness Scale (LEAS-J). Biopsychosoc Med. 2011;5, 2.

3. Huggins CF, Cameron IM, Williams JHG. Autistic traits predict underestimation of emotional abilities. J Exp Psychol Gen. 2021;150, 930–42.

4. Huggins CF, Cameron IM, Williams JHG. Different aspects of emotional awareness in relation to motor cognition and autism traits. Front Psychol. 2019;10, 2439.

5. Moriguchi Y, Maeda M, Igarashi T, Ishikawa T, Shoji M, Kubo C, Komaki G. Age and gender effect on alexithymia in large, Japanese community and clinical samples: A cross-validation study of the Toronto Alexithymia Scale (TAS-20). Biopsychosoc Med. 2007;1, 7.

6. Bagby RM, Parker JDA, Taylor GJ. The twenty-item Toronto Alexithymia scale—I. Item selection and cross-validation of the factor structure. J Psychosom Res. 1994;38, 23–32.

7. Komaki G, Maeda M, Arimura T, Nakata A, Shinoda H, Ogata I, Shimura N, Kawamura C, Kubo C. The reliability and factorial validity of the Japanese version of the 20-item Toronto Alexithymia Scale (TAS-20). Jap J Psychosom Med. 2003;43, 839–46.

8. Taylor GJ, Bagby RM, Parker JDA. The 20-item Toronto Alexithymia Scale. IV. Reliability and factorial validity in different languages and cultures. J Psychosom Res. 2003;55, 277–83.

9. Chan J, Becerra R, Weinborn M, Preece, D. Assessing alexithymia across Asian and Western Cultures: Psychometric properties of the Perth Alexithymia Questionnaire and Toronto Alexithymia Scale-20 in Singaporean and Australian samples. J Pers Assess. 2023;105, 396–412.

10. Tuliao AP, Klanecky AK, Landoy BVN, McChargue DE. Toronto Alexithymia Scale–20: Examining 18 competing factor structure solutions in a U.S. sample and a Philippines sample. Assessment. 2020;27, 1–17.

11. Marchewka A, Żurawski Ł, Jednoróg K, Grabowska A. The Nencki Affective Picture System (NAPS): Introduction to a novel, standardized, wide-range, high-quality, realistic picture database. Behav Res Methods. 2014;46, 596–610.

12. Erbas Y, Ceulemans E, Lee Pe M, Koval P, Kuppens P. Negative emotion differentiation: Its personality and well-being correlates and a comparison of different assessment methods. Cogn Emo. 2014;28, 1196–213.

13. Erbas Y, Ceulemans E, Blanke ES, Sels L, Fischer A, Kuppens P. Emotion differentiation dissected: between-category, within-category, and integral emotion differentiation, and their relation to well-being. Cogn Emo. 2019;33, 258–71.

14. Delacre M, Lakens D, Lays C. Why psychologists should by default use Welch’s t-test instead of student’s t-test. Int Rev Soc Psychol. 2017;30, 92–101.
