# Supplementary material for: Cross-cultural differences in self-reported and behavioural emotional self-awareness between Japan and the UK
Source: BMC Res Notes. 2023 Dec 21;16:380. doi: 10.1186/s13104-023-06660-0 (PMC10734098; doi:10.1186/s13104-023-06660-0)
Supplement: Supplementary file 2 — Supplementary Material 2 [file 13104_2023_6660_MOESM2_ESM.pdf]

Code and output for main analyses

Author 1

14/04/2021

This document shows the R code and output for all statistical analyses in the paper C*ross-cultural differences in emotional self-awareness between the UK and Japan.*

Data Preparation & Summary Statistics

Pull in raw data.

```
data = read_excel("2021-04-12 JPN Dataset.xlsx")

df <- data %>%

  filter(NativeLang < 3)

UK_only <- df %>%

  filter(NativeLang == 2)

JPN_only <- df %>%

  filter(NativeLang == 1)
```

Pull averages of self-report measures.

```
av_culture <- df %>%
  group_by(NativeLang) %>%
  get_summary_stats(
    TAS20, DIF, DDF, EOT, IRI, BAPQ, HADS, AFQ, TotalConsistency_LOG, PosMSD, NegMSD,
    type =  "common",
    probs = seq(0, 1, 0.25)
  )

formattable(av_culture)
```

NativeLang	variable	n	min	max	median	iqr	mean	sd	se	ci
1	AFQ	29	18.000	46.000	31.000	7.000	30.241	5.884	1.093	2.238
1	BAPQ	29	2.056	4.000	2.778	0.833	2.906	0.528	0.098	0.201
1	DDF	29	8.000	23.000	16.000	8.000	16.172	4.457	0.828	1.695
1	DIF	29	10.000	33.000	16.000	7.000	17.897	6.207	1.153	2.361

NativeLang	variable	n	min	max	median	iqr	mean	sd	se	ci
1	EOT	29	12.000	34.000	17.000	5.000	17.828	4.622	0.858	1.758
1	HADS	29	3.000	19.000	8.000	10.000	9.241	5.376	0.998	2.045
1	IRI	29	41.000	96.000	77.000	11.000	75.483	12.339	2.291	4.694
1	NegMSD	29	0.512	2.336	1.661	0.491	1.643	0.416	0.077	0.158
1	PosMSD	29	0.803	2.016	1.339	0.548	1.364	0.381	0.071	0.145
1	TAS20	29	37.000	72.000	49.000	18.000	51.897	11.169	2.074	4.248
1	TotalConsistency_LOG	29	1.398	2.640	2.004	0.270	2.003	0.269	0.050	0.102
2	AFQ	40	22.000	36.000	30.000	6.250	29.600	3.967	0.627	1.269
2	BAPQ	43	1.750	3.861	2.833	0.806	2.789	0.536	0.082	0.165
2	DDF	43	6.000	23.000	12.000	7.500	12.860	4.843	0.739	1.491
2	DIF	43	7.000	29.000	15.000	8.000	15.302	5.549	0.846	1.708
2	EOT	43	7.000	24.000	14.000	5.000	14.233	3.884	0.592	1.195
2	HADS	43	3.000	29.000	12.000	7.000	13.140	6.372	0.972	1.961
2	IRI	43	22.000	90.000	72.000	16.000	68.791	13.797	2.104	4.246
2	NegMSD	43	0.179	2.416	1.603	0.751	1.527	0.510	0.078	0.157
2	PosMSD	43	0.244	2.141	1.291	0.488	1.240	0.426	0.065	0.131
2	TAS20	43	26.000	71.000	42.000	16.500	44.442	11.654	1.777	3.587
2	TotalConsistency_LOG	43	0.954	2.504	1.813	0.387	1.782	0.333	0.051	0.103

Conduct t-tests to examine differences in total self-report measure scores between Japanese and UK cultural groups.

```
t.test(JPN_only$AFQ, UK_only$AFQ)
```

```
##
##  Welch Two Sample t-test
##
## data:  JPN_only$AFQ and UK_only$AFQ
## t = 0.50912, df = 45.913, p-value = 0.6131
## alternative hypothesis: true difference in means is not equal to 0
## 95 percent confidence interval:
##  -1.894572  3.177330
## sample estimates:
## mean of x mean of y
##  30.24138  29.60000
```

```
t.test(JPN_only$BAPQ, UK_only$BAPQ)
```

```
##
##  Welch Two Sample t-test
##
## data:  JPN_only$BAPQ and UK_only$BAPQ
## t = 0.91907, df = 60.834, p-value = 0.3617
## alternative hypothesis: true difference in means is not equal to 0
## 95 percent confidence interval:
##  -0.1380080  0.3727491
## sample estimates:
```

```
## mean of x mean of y
##   2.90613   2.78876
```

```
t.test(JPN_only$HADS, UK_only$HADS)
```

```
##
##  Welch Two Sample t-test
##
## data:  JPN_only$HADS and UK_only$HADS
## t = -2.7981, df = 66.432, p-value = 0.00672
## alternative hypothesis: true difference in means is not equal to 0
## 95 percent confidence interval:
##  -6.679322 -1.116990
## sample estimates:
## mean of x mean of y
##  9.241379 13.139535
```

```
t.test(JPN_only$IRI, UK_only$IRI)
```

```
##
##  Welch Two Sample t-test
##
## data:  JPN_only$IRI and UK_only$IRI
## t = 2.1512, df = 64.537, p-value = 0.03521
## alternative hypothesis: true difference in means is not equal to 0
## 95 percent confidence interval:
##   0.4784559 12.9056660
## sample estimates:
## mean of x mean of y
##  75.48276  68.79070
```

```
t.test(JPN_only$TAS20, UK_only$TAS20)
```

```
##
##  Welch Two Sample t-test
##
## data:  JPN_only$TAS20 and UK_only$TAS20
## t = 2.7293, df = 61.951, p-value = 0.00825
## alternative hypothesis: true difference in means is not equal to 0
## 95 percent confidence interval:
##   1.994801 12.914582
## sample estimates:
## mean of x mean of y
##  51.89655  44.44186
```

```
t.test(JPN_only$DIF, UK_only$DIF)
```

```
##
##  Welch Two Sample t-test
##
## data:  JPN_only$DIF and UK_only$DIF
## t = 1.8144, df = 55.557, p-value = 0.07502
## alternative hypothesis: true difference in means is not equal to 0
## 95 percent confidence interval:
##  -0.2705768  5.4590291
## sample estimates:
## mean of x mean of y
```

```
## 17.89655 15.30233
```

t.test(JPN_onlyDDF, UK_onlyDDF)

```
##
## Welch Two Sample t-test
##
## data: JPN_only$DDF and UK_only$DDF
## t = 2.9858, df = 63.507, p-value = 0.004014
## alternative hypothesis: true difference in means is not equal to 0
## 95 percent confidence interval:
## 1.095674 5.528224
## sample estimates:
## mean of x mean of y
## 16.17241 12.86047
```

t.test(JPN_onlyEOT, UK_onlyEOT)

```
##
## Welch Two Sample t-test
##
## data: JPN_only$EOT and UK_only$EOT
## t = 3.4474, df = 53.009, p-value = 0.001116
## alternative hypothesis: true difference in means is not equal to 0
## 95 percent confidence interval:
## 1.503384 5.686672
## sample estimates:
## mean of x mean of y
## 17.82759 14.23256
```

t.test(JPN_only$TotalConsistency_LOG, UK_only$TotalConsistency_LOG)

```
##
## Welch Two Sample t-test
##
## data: JPN_only$TotalConsistency_LOG and UK_only$TotalConsistency_LOG
## t = 3.1046, df = 67.684, p-value = 0.002782
## alternative hypothesis: true difference in means is not equal to 0
## 95 percent confidence interval:
## 0.07897547 0.36322348
## sample estimates:
## mean of x mean of y
## 2.002828 1.781728
```

t.test(JPN_only$PosMSD, UK_only$PosMSD)

```
##
## Welch Two Sample t-test
##
## data: JPN_only$PosMSD and UK_only$PosMSD
## t = 1.2908, df = 64.547, p-value = 0.2014
## alternative hypothesis: true difference in means is not equal to 0
## 95 percent confidence interval:
## -0.06787145 0.31586424
## sample estimates:
## mean of x mean of y
## 1.363601 1.239605
```

```
t.test(JPN_only$NegMSD, UK_only$NegMSD)

## 
##  Welch Two Sample t-test
## 
## data:  JPN_only$NegMSD and UK_only$NegMSD
## t = 1.0527, df = 67.375, p-value = 0.2963
## alternative hypothesis: true difference in means is not equal to 0
## 95 percent confidence interval:
##  -0.1033111  0.3339282
## sample estimates:
## mean of x mean of y 
##  1.642797  1.527488
```

Comparing Self-Reported Emotional Self-Awareness by Cultural Groups

Comparing how Japanese and UK participants scored on the self-report measure of emotional self-awareness, the TAS-20. Done using a linear rgeression to control for the effect of sex and age.

Recode binary variables into dummy variables.

```
#Recode gender
# 0 = M, 1 = F

df$Gender[df$Gender == 1] <- 0
df$Gender[df$Gender == 2] <- 1


#Recode Native language
# 0 = JPN, 1 = ENG
df$NativeLang[df$NativeLang == 1] <- 0
df$NativeLang[df$NativeLang == 2] <- 1
```

Run linear regression and standardise co-efficients.

```
## Linear regression
# Dependent: Total TAS-20 scores
# Independent: Nationality (Japanese vs UK)
# Covariates: Depression, Sex, and Age

model <- lm(TAS20 ~ as.factor(NativeLang) + as.factor(Gender) + Age, data=df)


summary(model)

## 
## Call:
## lm(formula = TAS20 ~ as.factor(NativeLang) + as.factor(Gender) + 
##     Age, data = df)
## 
```

```
## Residuals:
##     Min      1Q  Median      3Q     Max
## -17.956  -9.391  -1.937   9.075  25.518
##
## Coefficients:
##                         Estimate Std. Error t value Pr(>|t|)
## (Intercept)              60.0154     5.5421  10.829   <2e-16 ***
## as.factor(NativeLang)1   -7.6479     2.9056  -2.632   0.0105 *
## as.factor(Gender)1       -1.5254     2.9587  -0.516   0.6078
## Age                      -0.3130     0.2107  -1.486   0.1420
## ---
## Signif. codes:  0 '***' 0.001 '**' 0.01 '*' 0.05 '.' 0.1 ' ' 1
##
## Residual standard error: 11.42 on 68 degrees of freedom
## Multiple R-squared:  0.1272, Adjusted R-squared:  0.08873
## F-statistic: 3.304 on 3 and 68 DF,  p-value: 0.02532
```

```
lm.beta(model)
```

```
##
## Call:
## lm(formula = TAS20 ~ as.factor(NativeLang) + as.factor(Gender) +
##     Age, data = df)
##
## Standardized Coefficients::
##           (Intercept) as.factor(NativeLang)1     as.factor(Gender)1
##            0.00000000            -0.31576915            -0.06113445
##                   Age
##           -0.17039751
```

Also ran logistic regression assessing how TAS-20 subscales predicted cultural group while controlling for gender and age.

```
## Logistic regression of how TAS-20 scores predict group membership

model <- glm(as.factor(NativeLang) ~ as.factor(Gender) + Age + DIF + DDF + EOT, data=df, family="binomial")

tidy(model, conf.int=TRUE, exponentiate=TRUE)
```

```
## # A tibble: 6 x 7
##   term               estimate std.error statistic p.value conf.low conf.high
##   <chr>                 <dbl>     <dbl>     <dbl>   <dbl>    <dbl>     <dbl>
## 1 (Intercept)         224.       1.82       2.98  0.00293    7.83  10893.
## 2 as.factor(Gender)1    2.62     0.591      1.63  0.103      0.824     8.56
## 3 Age                   0.937    0.0420    -1.54  0.124      0.851     1.01
## 4 DIF                   0.986    0.0654    -0.223 0.824      0.863     1.12
## 5 DDF                   0.905    0.0849    -1.17  0.242      0.762     1.07
## 6 EOT                   0.855    0.0759    -2.06  0.0392     0.727     0.982
```

Comparing Behavioural Emotional Self-Awareness by Cultural Groups

Examining cultural group differences in EC-Task performance. Linear regression controlling for control condition scores, age and gender.

```
EC_model <- lm(TotalConsistency_LOG ~ NativeLang + Control_LOG + Gender + Age, data=df)
summary(EC_model)
```

```
##
## Call:
## lm(formula = TotalConsistency_LOG ~ NativeLang + Control_LOG +
##     Gender + Age, data = df)
##
## Residuals:
##      Min       1Q   Median       3Q      Max
## -0.77087 -0.16597  0.02385  0.18220  0.64752
##
## Coefficients:
##              Estimate Std. Error t value Pr(>|t|)
## (Intercept)  1.949422   0.167137  11.664  < 2e-16 ***
## NativeLang  -0.291074   0.075614  -3.849 0.000267 ***
## Control_LOG  0.150009   0.070624   2.124 0.037361 *
## Gender       0.144931   0.076866   1.886 0.063698 .
## Age         -0.008319   0.005455  -1.525 0.131974
## ---
## Signif. codes:  0 '***' 0.001 '**' 0.01 '*' 0.05 '.' 0.1 ' ' 1
##
## Residual standard error: 0.2956 on 67 degrees of freedom
## Multiple R-squared:  0.2226, Adjusted R-squared:  0.1762
## F-statistic: 4.797 on 4 and 67 DF,  p-value: 0.001833
```

```
lm.beta(EC_model)
```

```
##
## Call:
## lm(formula = TotalConsistency_LOG ~ NativeLang + Control_LOG +
##     Gender + Age, data = df)
##
## Standardized Coefficients::
## (Intercept)  NativeLang Control_LOG      Gender         Age
##   0.0000000  -0.4413521   0.2303283   0.2133187  -0.1663228
```

Examined whether PED-Task performance differed by nationality, controlling for gender and age. MANOVA used to assess both positive and negative differentiation scores.

```
PED_model <- manova(cbind(NegMSD, PosMSD) ~ NativeLang + Age + Gender, data = df)
summary(PED_model)
```

```
##            Df   Pillai approx F num Df den Df Pr(>F)
## NativeLang  1 0.028293  0.97542      2     67 0.3823
## Age         1 0.063143  2.25786      2     67 0.1125
## Gender      1 0.005304  0.17862      2     67 0.8368
## Residuals  68
```

```
summary.aov(PED_model)
```

```
##  Response NegMSD :
##             Df  Sum Sq  Mean Sq F value Pr(>F)
```

```
## NativeLang   1  0.2303 0.230281  0.9949 0.3221
## Age          1  0.0013 0.001264  0.0055 0.9413
## Gender       1  0.0053 0.005335  0.0230 0.8798
## Residuals   68 15.7394 0.231462
##
##  Response PosMSD :
##             Df  Sum Sq Mean Sq F value  Pr(>F)
## NativeLang   1  0.2663 0.26629  1.6478 0.20361
## Age          1  0.6622 0.66223  4.0980 0.04686 *
## Gender       1  0.0388 0.03877  0.2399 0.62585
## Residuals   68 10.9887 0.16160
## ---
## Signif. codes:  0 '***' 0.001 '**' 0.01 '*' 0.05 '.' 0.1 ' ' 1
```

Plot of ESA Scores

Create violin plot of different ESA measures between cultural groups.

```
TAS.violin <- ggplot(df, aes(x = as.factor(NativeLang), y = TAS20, fill=as.factor(NativeLang))) +
  geom_violin(position=position_dodge(1)) +
  geom_boxplot(width = .1) +
  labs(x = " ", y="Total TAS-20 Score\n", caption="A: Higher scores indicate poorer emotional self-awareness\n") +
  scale_fill_manual(values=(c("#FFDE7B", "#6CA8DC"))) +
  scale_x_discrete(labels=c("Japan", "UK")) +
  theme_classic() +
  theme(text = element_text(size = 12, color="black")) +
  theme(plot.caption = element_text(face = "italic", size = 8)) +
  theme(panel.spacing = unit(0, "lines")) +
  theme(legend.position="none")

EC.violin <- ggplot(df, aes(x = as.factor(NativeLang), y = TotalConsistency_LOG, fill=as.factor(NativeLang))) +
  geom_violin(position=position_dodge(1)) +
  geom_boxplot(width = .1) +
  labs(x = " ", y="Total Inconsistency Score\n", caption="B: Higher scores indicate poorer emotional self-
awareness\n") +
  scale_fill_manual(values=(c("#FFDE7B", "#6CA8DC"))) +
  scale_x_discrete(labels=c("Japan", "UK")) +
  theme_classic() +
  theme(text = element_text(size = 12, color="black")) +
  theme(plot.caption = element_text(face = "italic", size = 8)) +
  theme(panel.spacing = unit(0, "lines")) +
  theme(legend.position="none")

pos.violin <- ggplot(df, aes(x = as.factor(NativeLang), y = PosMSD, fill=as.factor(NativeLang))) +
  geom_violin(position=position_dodge(1)) +
  geom_boxplot(width = .1) +
  labs(x = " ", y="Positive Differentiation Score\n", caption="C: Higher scores indicate better emotional self-
awareness\n") +
  scale_fill_manual(values=(c("#FFDE7B", "#6CA8DC"))) +
  scale_x_discrete(labels=c("Japan", "UK")) +
  theme_classic() +
  theme(text = element_text(size = 12, color="black")) +
  theme(plot.caption = element_text(face = "italic", size = 8)) +
  theme(panel.spacing = unit(0, "lines")) +
```
